# Dental Operations for Children*One of a symposium of papers read by women dentists at the Ohio State Dental Society, Dec., 1918. Reprinted in part from *Dental Summary* for February, 1919.

**Published:** 1919-03

**Authors:** Flora N. Haag


### DENTAL OPERATIONS FOR CHILDREN*

BY FLORA N. HAAG, D.D.S.

This is the age of prevention in dentistry as well as in medicine, and he who fully realizes this important fact must, of necessity, adopt as his watchword that important term *Preservation*, with all that the word implies. Restorative procedures always will be necessary, but their importance gradually will decrease with the advance of scientific knowledge in our profession.

There is no work which is of greater importance or attended with greater difficulties than that incident to the performance of dental operations upon the deciduous teeth; and there is no work connected with our profession that, in the long run, gives greater results. This work is more troublesome and requires more patience and skill than that done on the permanent teeth; yet the self-satisfaction of work well done at this opportune time more than pays. Many dentists do not care to treat children; they look upon this phase of their profession as temporizing. To be able to know how to perform successful operations that will preserve the temporary teeth until the time that they should be lost, and which will prevent future ills and give to the child a healthy oral cavity, that he may go through life with the number of teeth that nature intended that he should have, is by no means a menial service.

The necessary qualifications for the dentist who aspires to practice among children must be of a high order. He must possess unusual professional attainments, a knowledge of human nature, extreme kindness, patience, perseverance, tact, sympathy, firmness and a knowledge of the proper treatment to be given. Painful operations

* One of a symposium of papers read by women dentists at the Ohio State Dental Society, Dec., 1918. Reprinted in part from *Dental Summary* for February, 1919.

should be avoided if possible, certainly upon the first visit. Perfect operations are not to be gained at the expense of serious mental impressions made on the child. When a child enters a dental office for the first time and is introduced to the dentist, it is much the same as when two pugilists enter the ring. There is an appraisal on the part of both. The dentist is curious to know just how the little patient is going to receive him. He notes each movement and word; he tries to read the thoughts of the little one that he may know the best way in which to obtain the control that will enable him to do that which is best for the child. The child, a bundle of impulses, quick to think, already frightened at the thought of pain, looks the monster over and wonders where he is going to strike first. Even a pleasant greeting and friendly conversation will not always dispel the attitude of wonder and excited expectation. The same method of procedure can not be applied in every case, but as a rule, children should be treated like little men and women. It always is best, if possible, to avoid suspicion on the part of the child that the dentist suspects he is afraid. It is never wise to conceal instruments, but rather an explanation of their use should be made, if the child is curious to know. Deception of any kind never should be practiced, for, once having been deceived, the child's cooperation never will be reestablished and its confidence in humanity will be lessened.

Parents often tell the child that certain operations will not hurt. The dentist who is courageous and has the welfare of the little patient at heart, immediately will correct this statement, if the operation in hand is of such a nature that it can not be done without some pain. A personal interest in the child should be manifested, and his powers of resistance never should be overtaxed.

So important is the care of the oral cavity before the eruption of the deciduous teeth and during dentition, that it is necessary to employ some method by which parents may be taught the proper care in providing for a normal dentition. Many parents are ignorant of the evils of thumb-sucking and pacifiers, and even encourage them. It is not only after, but also preceding the eruption of the temporary teeth that mouth-breathing and thumb-sucking should be prevented. Place the thumb between the arches and it readily may be seen that as a result of this habit the upper arch will be forced outward, while the lower will be carried inward, producing an abnormal relationship of both arches.

Hygiene of the oral cavity should begin at birth. The nurse by means of a sterile napkin wrapped around the index finger, may cleanse the mouth by the use of distilled water or a lotion of boracic acid. Daily hygiene of the mouth not only keeps the tissues healthy, but it soon accustoms the child to expect it and helps to form the habit. As soon as the child is old enough, it may be given a fine bristle brush or camel's hair brush and taught how to use it. It also should be given thorough instruction as to why its mouth should be kept clean.

The three crucial periods in the life history of an individual are the periods of dentition, puberty and the menopause, and of these three I do not hesitate to say that dentition is the one fraught with the greatest danger and requiring the most painstaking care on the part of both physician and patient. The period of dentition usually is one of great annoyance to the child. There is a diversity of opinion among physicians in regard to the many ills that usually arise during the teething period, and the advisability of lancing has been questioned. We do know that during the first and second summers improper feeding is the cause of various diseases attributed to teething. It is known also, that a twenty-months-old child slept for hours after a gum over a molar tooth was lanced; also, a child with a temperature of 103 degrees became normal in a few hours after the performance of a similar operation. The pain, however, is not caused altogether by the gum overlying the tooth; but when the organ is bound down by the dense gum above it, the growth of the root presses upon the formative organ below, often causing disturbances of an alarming character.

The fact of the matter is that pain and other pathological disturbances arising from tooth eruption originate from four different sources, viz.: the overlying gum, the tissues at the end of the developing root, the tooth pulp and the peridental membrane.

In lancing the gum, the more successful operations have been where the overlying tissues have been dissected away from that portion of the crown which is being held in bondage. A simple straight incision across the occlusal surface or incisal edge does not suffice, as the gum tissue heals rapidly and soon will repair itself and the desired results will not have been obtained.

There are many important reasons for preserving the temporary teeth, prominent among which are: avoidance of pain, preventing bad habits of mastication and shaping a symmetrical jaw. The voluntary disuse of the teeth frequently is a direct result of caries, which renders the chewing of food difficult and painful, with the result that food is swallowed without proper mastication, and the habit of bolting the food is formed and carried through life, causing many systemic derangements. Many practitioners appreciate the value of preserving the temporary teeth from the masticatory and succedaneous view points; but they do not realize all of the important functions which the deciduous teeth perform, and will extract a decayed deciduous tooth without compunction, failing to realize that the child, nevertheless, is more or less injured for life. The preservation of the temporary teeth assists in the growth and development of the jaw to the extent that it permits the proper eruption and alignment of the permanent teeth.

Having had thirteen years' practice, seven of which were in a college where many children were treated daily, I did not fully realize the injury resulting from a lost deciduous tooth if it had served part or most of its time until I became associated with Dr. F. M. Casto, orthodontist. The loss of a temporary tooth by decay, inflammation of the pulp or abscess, not only causes much pain and suffering, not only interferes with the nervous system, not only prevents proper mastication, not only disturbs the digestive function at a time when it is so important to the child's growth, not only interferes with the maintenance of proper hygienic surroundings, but it also lessens the size of the already small arch, instead of assisting in its development and growth.

The maintenance of the normal mesio-distal width is also of great importance. The loss of the temporary molars will allow the first permanent molars to move forward and take up the space that later should be occupied by the bicuspids. The normal eruption of the permanent denture depends largely upon the first permanent molar erupting in its proper position. If the pulp of a deciduous tooth is diseased, there will be an interference with the absorption of the root, thereby disturbing the eruption of the permanent tooth.

The most difficult operations upon the deciduous teeth are the devitalization of pulps, the filling of root canals, and the treatment of abscesses. The medicaments to be used for the devitalization of the pulp will depend upon the amount of absorption of the root. There is a marked difference in the time of root absorption in different individuals and in the individual teeth of the same person; but the average time of beginning absorption of the root of the first deciduous molar is seven years of age, while that of the second usually is eight, and the cuspid, nine.

Up until this time any method of devitalization used in the permanent may be used with safety in the temporary teeth. If any absorption of the root has begun, arsenic trioxid should not be used. If there is any doubt in the mind of the operator as to the conditions present, an X-ray should be made of the root. Novocain hydrochlorid pressure anesthesia is used by many operators with success, although some dentists are of the opinion that should the root be partially absorbed, the anesthetic might be carried into the soft tissues and dangerous results arise. However, a very small amount is necessary to anesthetize the pulp in a deciduous tooth, and when used with the proper caution, evil effects never have been noted in the writer's practice. Phenol, under pressure, frequently is an efficient and safe method for pulp extirpation, since there is no systemic effect and it limits its own action. This method has brought marked results. Infiltration also may be used with good results in some cases, while conductive anesthesia is contraindicated.

In the filling of the root canals of deciduous teeth the same care should be observed as in filling permanent roots. However, it is best to fill temporary roots, and especially those which are partially absorbed, with a filling material which will absorb with the roots.

Dr. W. H. O. McGehee, in his manuscript of a text book on Operative Dentistry soon to be issued, mentions the following materials which may be used for this purpose, viz.:

(*a*) Salol, liquified by heat and pumped into the canals with a smooth broach;

(*b*) Salol and aristol, equal parts, used in the same manner;

(*c*) Salol, aristol and paraffin, same method;

(*d*) Paraffin, melted and pumped into the canals;

(*e*) Prinz' root-canal filling;

(*f*) Balsamo del deserto;

(*g*) Zinc oxid or calcium oxid made into a paste with oil of cloves, eugenol or formocresol;

(*h*) Euca-percha (Buckley).

In regard to the treatment of alveolar abscess in deciduous teeth he also gives the following:

"The treatment for alveolar abscess in temporary teeth is practically the same as for permanent teeth, as far as conditions permit. If the tooth is very sore, the passage of a broach into the canals, after flushing out the cavity, often will afford exit for malodorous gases and other products of putrefactive decomposition and relieve the acute symptoms. Lancing of the gum is practiced under the same circumstances as indicated for permanent teeth, and often affords quick relief from pain. As soon as conditions will allow, the chamber and canals may be opened and a modified formocresol treatment applied. These cases usually respond much more readily to treatment than permanent teeth."

Dr. Black mentions the fact that he has observed a number of cases, one in his own family, where an abscess occurred at the end of a root of a temporary molar before the enamel of the crown of the bicuspid had been completed, and, in that case, the pus had broken through into the enamel organ and destroyed it, or a part of it, so that the enamel for the crown of the bicuspid never was completed. Then it came through as an imperfectly-enameled tooth. He adds that a severe alveolar abscess that may occur early at the root of a deciduous molar, may be relieved by discharge of pus or the removal of the offending tooth, and be forgotten. Then the bicuspid takes its place, with imperfect enamel, and no one knows what has occurred. A number of cases had occurred in his practice in which necrosis, as a result of these abscesses had brought away the permanent tooth with that portion of bone immediately surrounding it. This leads one to believe that the sufferings of children from these conditions are not sufficiently appreciated, and it is certain that the little ones are neglected much too often.

We find dental caries beginning in teeth as early as two years of age. It is as painful to prepare cavities in the teeth for the little ones as it is for the adult, and perhaps more so. Where the cavities are small, we may fill the teeth with the same materials as are used for the permanent teeth. Where cavities are large in molars, we may not always be able to make the proper excavation. In these cases silver nitrate will aid in relieving the sensitiveness and also in arresting decay. After a few applications we will find that we will be able to remove the majority of decay and make a filling.

The occlusal surfaces of the molar should be opened very wide in order that the filling may extend beyond the sulci, for the purpose of preventing food from packing in and fermenting, thus preventing recurrence of decay. When silver nitrate is used, care should be taken not to have the tooth blackened near the margin. The improved copper cement, amalgam, or synthetic, should be used for the filling of the occlusal surfaces of the molars. If the cavities are deep-seated a guttapercha intermediate should first be placed on the floor of the cavity and covered with cement. The proximal surfaces of the deciduous molars are extremely difficult to handle. Owing to the extreme sensitiveness of the proximal cavity, the gum which approaches very close to the contact point, and the large size of the pulp, we usually find the preparation of these cavities a strenuous task if we succeed in making a perfect operation. We find it necessary to exercise the greatest care and skill in the preparation of these cavities because of the danger of encroaching upon the pulp. When small, we usually find the decay close to the occlusal portion of the surface. These cavities frequently may be prepared without entirely destroying the contact point of the tooth. Amalgam is the best filling material to be used. A filling never should be bridged to a neighboring tooth. These fillings should be as carefully made, contoured and polished as the fillings for the permanent teeth, on account of the necessity for the protection of the gum tissue, the maintenance of the mesio-distal width and the preservation of the tooth. The difficulty is that parents usually do not bring their children until decay has gone far enough to cause toothache. Then the first effort should be to relieve the pain, and nothing more should be done until a subsequent sitting. If the pulp is exposed, which frequently is the case, it may be devitalized, the roots filled and the crown filling made.

The first permanent molar which usually erupts very quietly, is often mistaken by the parent for a temporary tooth and for that reason frequently is neglected. All dentists however should appreciate the importance of this tooth in the arch. These usually are the first of the permanent teeth to be attacked by decay, and if decay has not started, it may be prevented by cleansing the occlusal surface and flowing into the pits and fissures some improved copper cement. Where decay has progressed, the gold inlay makes a splendid filling. Where cavities are small, synthetic is frequently the most desirable material to be used.

### THE DENTIST'S AND PARENT'S RESPONSIBILITY

Sad though it may seem, it is true that many parents not only are ignorant of the necessity of preserving the temporary teeth, but are too busy with social functions or the accumulation of wealth, to look after the proper care and training of their children. The failure of the dentist to teach the parent that the neglect of the proper training of the young child that it may grow up strong mentally and physically, allowing disease to enter the body when it could be prevented by proper care is criminal, and parents should be made to understand that they, with the dentist, are held responsible for the welfare of the child. It is possible that parents do not realize that in the interest of their children a healthy body is more conducive to happiness than the inheritance of great wealth.

Dentists should see to it that they instruct mothers to teach their children to acquire habits of thorough mastication and daily cleansing, the use of the tooth brush, the necessity of caring for the temporary teeth, the bad effect of bolting the food, the need of correcting abnormal conditions about the teeth and the repair of each individual tooth, whether it be deciduous or permanent, to visit the dentist early and often before the need seems apparent, as well as the necessity for preserving the first permanent molar.

The American flag is the symbol of liberty, not only to our own country but to all the countries of the world. Parents teach their children this fact for the purpose of thrilling their young minds with ideas of patriotism and loyalty. This is right; but are parents and dentists wholly loyal and patriotic if they do not use every means within their power to give to their country men and women with healthy bodies and healthy minds? Early in life the child is dependent on others; the dentist and parents stand guard at the gateway of health, and, if having slept on duty, the enemy enters, the body is impaired and the system is poisoned.

If one will take care of his teeth when he is young, I'm told,
His teeth will take good care of him when he is getting old.

